# Kallikrein 12 Regulates Innate Resistance of Murine Macrophages against *Mycobacterium bovis* Infection by Modulating Autophagy and Apoptosis

**DOI:** 10.3390/cells8050415

**Published:** 2019-05-05

**Authors:** Naveed Sabir, Tariq Hussain, Yi Liao, Jie Wang, Yinjuan Song, Muhammad Shahid, Guangyu Cheng, Mazhar Hussain Mangi, Jiao Yao, Lifeng Yang, Deming Zhao, Xiangmei Zhou

**Affiliations:** 1 Key Laboratory of Animal Epidemiology and Zoonosis, Ministry of Agriculture, National Animal Transmissible Spongiform Encephalopathy Laboratory, College of Veterinary Medicine, China Agricultural University, Beijing 100193, China; naveedsabir@upr.edu.pk (N.S.); drtariq@aup.edu.pk (T.H.); liaoyi_cau@126.com(Y.L.); wangjie1985abc@163.com (J.W.); syinjuan@126.com (Y.S.); hnchdd@163.com (G.C.); drmazharmangi114@gmail.com (M.H.M.); mole-yao@hotmail.com (J.Y.); yanglf@cau.edu.cn (L.Y.); zhaodm@cau.edu.cn (D.Z.); 2 Department of Clinical Veterinary Medicine, College of Veterinary Medicine, China Agricultural University, Beijing 100193, China; drshahid_vet@yahoo.com

**Keywords:** *Mycobacterium bovis*, murine macrophages, Kallikrein12, autophagy, apoptosis, cytokines

## Abstract

*Mycobacterium bovis* (*M. bovis*) is a member of the *Mycobacterium tuberculosis* (*Mtb*) complex causing bovine tuberculosis (TB) and imposing a high zoonotic threat to human health. Kallikreins (KLKs) belong to a subgroup of secreted serine proteases. As their role is established in various physiological and pathological processes, it is likely that KLKs expression may mediate a host immune response against the *M. bovis* infection. In the current study, we report in vivo and in vitro upregulation of KLK12 in the *M. bovis* infection. To define the role of KLK12 in immune response regulation of murine macrophages, we produced KLK12 knockdown bone marrow derived macrophages (BMDMs) by using siRNA transfection. Interestingly, the knockdown of KLK12 resulted in a significant downregulation of autophagy and apoptosis in *M. bovis* infected BMDMs. Furthermore, we demonstrated that this KLK12 mediated regulation of autophagy and apoptosis involves mTOR/AMPK/TSC2 and BAX/Bcl-2/Cytochrome c/Caspase 3 pathways, respectively. Similarly, inflammatory cytokines IL-1β, IL-6, IL-12 and TNF-α were significantly downregulated in KLK12 knockdown macrophages but the difference in IL-10 and IFN-β expression was non-significant. Taken together, these findings suggest that upregulation of KLK12 in *M. bovis* infected murine macrophages plays a substantial role in the protective immune response regulation by modulating autophagy, apoptosis and pro-inflammatory pathways. To our knowledge, this is the first report on expression and the role of KLK12 in the *M. bovis* infection and the data may contribute to a new paradigm for diagnosis and treatment of bovine TB.

## 1. Introduction

Tuberculosis (TB) is a major health threat to human and animal populations causing high morbidity and mortality worldwide. In 2017, about 10 million people developed active TB globally and 1.3 million deaths from TB were reported [1]. The disease is caused by the *Mycobacterium tuberculosis* complex (MTC) and *M. bovis* is an important member of MTC which is genetically more than 99% identical to *M. tuberculosis* [2]. *M. bovis* can cause TB in human and multiple species of animals including cattle with a similar disease profile and host immune response. It is estimated that it infects more than 50 million cattle per annum with global economic losses of approximately $3 billion [3]. *M. bovis* is responsible for 2.8% of all human TB cases in Africa and it also accounts for 7.6% of human TB cases in Mexico [4] while in a more recent study in Mexico, 30.2% of human TB was caused by *M. bovis* [5]. In a study in China, a total of 245 isolates from human TB were investigated and one isolate was identified as *M. bovis* [6]. Recently, a review addressing the epidemiology of human TB in the United States attributed 1.3 to 1.6% annual cases of human TB to *M. bovis* during the years 2006–2013 [7]. MTC members are intracellular pathogens and the immune response against these bacteria is primarily dependent on cellular immunity involving T lymphocytes and macrophages [8]. Macrophages act as a first line of defense by recognizing the invading mycobacteria through various pattern recognition receptors (PRRs). Activation of macrophages leads to induction of various protective mechanisms to restrain the *M. bovis* infection. 

Autophagy is an intracellular degradation process whereby cytosolic macromolecules and pathogens are transferred to lysosomes for degradation and removal from the cell [9]. The role of autophagy is well established in reducing the intracellular mycobacterial burden [10,11]. In an in vivo study, *Atg5* (autophagy-related protein 5) deficient mice were found to be more prone to TB as compared to the control group [12]. These findings elucidate the pivotal role of autophagy in curtailing Mtb associated damage to the host tissues. Similarly, the *M. bovis* infection can also trigger autophagy in mouse-derived macrophages [13,14]. Apoptosis is another host defense mechanism and it has been discovered that apoptosis and autophagy can take place concurrently in the same infected cell [15] and both mechanisms can share common signaling pathways [16,17]. 

Macrophages also produce inflammatory cytokines and present bacterial peptide to T lymphocytes [18]. Cytokines are small proteins that are produced by host cells and primarily act in paracrine fashion to regulate the function of adjacent cells. Almost every nucleated cell can produce and respond to cytokines indicating the key role of cytokines in homeostasis [19]. Cytokines such as IL-1β, IL-6, IL-12 and TNF-α are pro-inflammatory and mediate a protective immune response against the pathogen [20,21,22] while IL-10 is considered as an anti-inflammatory cytokine suppressing macrophage and dendritic cell (DC) function [23]. Recent reviews show that IFN-β also plays a pro-bacterial role by antagonizing the production and function of IL-1β and IL-18 [24,25]. Furthermore, TNF-α, IL-1, IL-6 and IL-10 have been shown to regulate autophagy [26]. These findings suggest that all of the events in the cellular immune response are complex and inter-related. It is well established now that mycobacterial infection of host immune cells leads to the differential expression of a plethora of genes in these cells. In a previous study by our lab [27], thousands of genes were differentially expressed in bovine macrophages upon an in vitro challenge with *M. bovis*. In the macrophages from the *M. bovis* infected animals, kallikrein-related peptidase 12 (KLK12) and other proteases were also differentially expressed.

Kallikreins (KLKs) are a subfamily of serine proteases primarily acting as enzymes involved in the cleavage of vasoactive peptides (kininogens into kinins) and they are classified as KLK1 to KLK15 [28,29]. Primarily, the KLK family has two isoforms: One is plasma KLK, which is produced in the pancreas and found in the blood circulation, and the other is tissue KLK, which is expressed and found in various tissues of the body. In this research article, KLKs is meant for tissue KLKs. Studies have revealed that KLKs genes are found in a human and also in many other animal species. In the case of a mouse, *KLKs* genes are located on the mouse chromosome 7 close to the Tam-1 locus. Human/mouse homology maps (http://www.ncbi.nlm.nih.gov/Homology/) show that this region of the mouse is highly similar to that of human chromosome 19q13.4 locus carrying human *KLK* genes and share 70–90% homology at mRNA and protein levels [28]. KLKs play a key role in various homeostatic and pathophysiological processes in the body [30,31]. They play a vital role in digestion, coagulation and fibrinolysis, inflammation, tissue remodeling, activation of hormones, growth factors production, receptors activation and extracellular matrix protein degradation. Their role in carcinogenesis is also well established and KLK3, also known as prostate specific antigen (PSA), is the most commonly used biomarker in prostate cancer [32]. In recent years, many studies have reported KLKs expression profiles and their role in the development and metastasis of various malignancies in humans [33,34,35,36]. It is believed that KLKs are involved in the pathogenesis of diabetes, renal disease, peripheral ischaemia and aldosterone-salt induced hypertension [37]. Some studies have shown that KLKs have neuroprotective effects in conditions of oxygen and glucose deprivation, and AMPK signaling takes part in these neuroprotective mechanisms of KLKs [38,39]. Liu et al. [40,41] reported that KLKs promote the survival of SH-SY5Y neuronal cells under nutrient deprivation conditions through induction of autophagy. Recently, many studies have explored the role of various KLKs in different diseases. For example, KLK8 proteolytically facilitates human papillomaviruses entry into host cells [42] and its inhibition can mitigate Alzheimer’s disease pathogenesis in mice [43]. Similarly, KLK5 also plays a role in the H3N2 influenza virus infection in humans [44] and KLK6 activates autophagy in various gastric cancer cell lines and mediates chemotherapeutic resistance by attenuating auranofin-induced cell death [45]. Expression of KLK12 and its role has been validated in breast and gastric cancers, and in the degradation of matricellular proteins [46,47,48]. 

As discussed above, a previous report by our lab [27], KLK12 was differentially expressed in bovine macrophages upon an in vitro challenge with *M. bovis* and no study has addressed the association between the KLK12 expression and *M. bovis* infection. Therefore, keeping in view the previous findings, the current study was designed to investigate in vivo and in vitro expression of KLK12, and its role in the regulation of autophagy in *M. bovis* infected murine macrophages. Furthermore, the effect [or effects] of KLK12 on apoptosis and cytokines expression was also studied as all these immune response mechanisms are inter-related and collectively enhance the innate resistance of the immune cells against invading pathogens. The current study revealed that the expression of KLK12 was significantly increased in the lungs and spleen of *M. bovis* infected mice. In vitro studies using bone marrow derived macrophages (BMDMs) and RAW264.7 macrophages also depicted an upregulated expression of KLK12 after the *M. bovis* infection. To determine the role of KLK12 in the regulation of an *M. bovis* induced autophagy, we produced KLK12 knockdown BMDMs by using the siRNA transfection technique. The knockdown of KLK12 resulted in significantly downregulating autophagy in *M. bovis* infected BMDMs. Furthermore, we demonstrated that this KLK12 regulation of autophagy involves the mTOR/AMPK/TSC2 pathway. Similarly, apoptosis and inflammatory cytokines were also downregulated implicating an important role of KLK12 in innate immunity against the *M. bovis* infection.

To our knowledge, this is the first study elaborating the expression of KLK12 and its role in the modulation of the innate immune response against the *M. bovis* infection. The study will also help to better understand the interaction between host immune cells and the invading pathogen. The study also opens a new avenue for diagnosis and a host directed therapy of the disease.

## 2. Materials and Methods

### 2.1. Ethics Statement

All the animal experiments were carried out in accordance with the Chinese Regulations of Laboratory Animals—The Guidelines for the Care of Laboratory Animals (Ministry of Science and Technology of People’s Republic of China) and Laboratory Animal Requirements of Environment and Housing Facilities (GB 14925–2010, National Laboratory Animal Standardization Technical Committee). The experiments involving the animal model were also duly approved by The Laboratory Animal Ethical Committee of China Agricultural University and the license number of the research protocol was CAU20171011–2. 

### 2.2. Reagents and Antibodies

The mouse KLK12 gene sequence was obtained from NCBI Gene Bank (NCBI Reference Sequence: NC_000073.6) and KLK12 primers were designed by using the Primer 3 software (Free Software Foundation, Franklin, Boston, MA, USA). All the primers used in the current study including *KLK12, β-actin, IL-1β, IL-6, IL-10, IL-12, TNF-α* and *IFN-β* were commercially prepared by the Genewiz Technology. Lipofectamine 3000 was from Invitrogen Thermo Fisher Scientific (Carlsbad, CA, USA), 7H9 Middlebrook media from Difco and mycobactin J from Dickinson and Sparks Company (Franklin Lakes, NJ, USA). The macrophage colony-stimulating factor (M-CSF) was purchased from Peprotech Technology. The rabbit polyclonal anti-KLK12 antibody (3720-100) was purchased from BioVision (BioVision Incorporated, Milpitas, CA, USA), Rabbit polyclonal alpha Tubulin antibody (11224-1-AP), Rabbit polyclonal β-Actin antibody (20536-1-AP), Rabbit polyclonal anti-LC3 antibody (18725-1-AP), Rabbit polyclonal Beclin 1 antibody (11306-1-AP), Rabbit polyclonal P62 antibody (18420-1-AP), Rabbit polyclonal β-Catenin antibody (51067-2-AP), Rabbit polyclonal BAX antibody (50599-2-Ig), Rabbit polyclonal Cytochrome C antibody (10993-1-AP), Rabbit polyclonal TOM20 antibody (11802-1-AP), peroxidase-conjugated goat anti-rabbit antibody (SA00001-2) were purchased from Proteintech (Wuhan, Hubei, China). Rabbit monoclonal Phospho-AMPKα (877-616-CELL), Rabbit monoclonal Phospho-mTOR antibody (S2481) and Rabbit monoclonal Phospho-Tuberin/TSC2 (877-678-CELL) were purchased from Cell Signaling Technology (Danvers, MA, USA). The rabbit polyclonal caspase 3 antibody (GR64557-1) was purchased from Abcam (Cambridge, UK) while the mouse monoclonal Bcl-2 antibody (F0106) was from Santa Cruz Biotechnology (Santa Cruz, CA, USA). The goat anti-mouse secondary antibody (ZB-5305) was obtained from Beijing ZSGB Biotechnology (Beijing, China). Mouse Il-1β, IL-10, IL-12 and IFN-β ELISA kits were purchased from Neobioscience Technology (Shenzhen, Guangdong, China). CellTiter 96Aqueous one solution cell proliferation assay kit was from Promega technology, USA. Rapamycin (Sirolimus) (S1039) was purchased from Selleckchem.com and bafilomycin A1 (A8510) was procured from Solarbio Life Sciences (Beijing, China). 

### 2.3. Bacterial Culture

In the current study, we used two virulent strains of the *M. bovis* C68004 strain was obtained from the China Institute of Veterinary Drug Control and this strain has been used for years with stable virulence. The *M. bovis* N strain was isolated recently from the brain tissue of cattle with generalized bovine tuberculosis. Both strains have been identified as *M. bovis* by the multilocus sequence typing analysis as described earlier [49] and the virulence of both strains have been reported recently [50]. The *M. bovis* N strain was isolated by our laboratory and cultured for few passages before infecting the cells while the *M. bovis* C68004 strain was obtained from cold storage and was passaged for three times after its re-isolation from experimentally infected animals. Both strains were grown in a 7H9 Middlebrook media (Difco) supplemented with 10% *v*/*v* OADC (Oleic acid, Albumin, Dextrose, Catalase) enrichment solution (BD Biosciences), 2 g/L sodium pyruvate and 0.05% Tween-80, and incubated at 37 °C under biosafety level 3 (BSL3) facilities, China Agricultural University, Beijing.

### 2.4. Mice and M. bovis Infection

Six to eight week old C57BL/6 mice were procured from Vital River Laboratories (Beijing, China). The mice were kept in the biosafety level 3 (BSL3) facilities, College of Veterinary Medicine, China Agricultural University, Beijing. For the *M. bovis* infection, mice were distributed into two groups having equal numbers of mice (*n* = 10). One group was intranasally inoculated with *M. bovis* C68004 at a dose rate of 200 CFU/mouse while the second group was inoculated with sterile phosphate buffered saline (PBS) via the same route. Mice from the both groups were sacrificed at the indicated time points. Lungs and spleen tissues were collected and processed to determine the expression of KLK12. 

### 2.5. Cell Cultures

RAW264.7 macrophages were obtained from the Cell Culture Center, Xiehe Medical University (Beijing, China) and were stored at −80 °C. The macrophages were taken from cold storage (−80 °C) and cultured in a humidified incubator at 37 °C with 5% CO_2_ in DMEM (Hyclone, Logan, UT, USA) supplemented with 10% FBS (Gibco, Grand Island, NY, USA), 100 μg/mL streptomycin and 100 U/mL penicillin (Gibco). Then, the macrophages were moved to 6 or 12-well cell culture plates for 12–18 h prior to transfection and/or infection. Previously reported protocols were followed for isolation and culture of mouse bone marrow-derived macrophages (BMDMs) [51]. Briefly, femurs and tibia of 6 to 8 weeks-old female C57BL/6 mice were used to sequester undifferentiated bone marrow cells. The cells were cultured in a RPMI1640 (Hyclone) media supplemented with 10% FBS, penicillin 100 U/mL and streptomycin 100 microgram/mL (Gibco) and macrophage colony stimulating factor (M-CSF) 10 ng/mL (Pepro Tech, Rocky Hill, NJ, USA). These BMDMs were cultured in cell culture flasks (Corning) for 7–8 days. Then, the adherent BMDMs (6–8 × 10^6^ cells) were transferred to 6 or 12-well cell culture plates for infection and/or transfection experiments.

### 2.6. Cells Transfection and Infection

BMDMs and RAW264.7 macrophages were allowed to attach overnight in 6 or 12-well plates (2 × 10^5^ cells in each well). The following day, three KLK12 siRNAs with different sequences, one control, and one FAM negative control (Synbio Technology, China) were individually transfected (20 nM) into RAW264.7 and BMDM cells using Lipofectamine 3000 reagent (Invitrogen, Carlsbad, CA, USA) according to the manufacturer’s instructions. The sequences of three KLK12 siRNAs, negative control siRNA and FAM siRNA negative control are shown in table (Supplementary Table S1). After 6 h of transfection, the original medium was replaced with fresh medium supplemented with 10% FBS. After 48 h post-transfection, FAM negative control treated macrophages were observed under fluorescent microscope to examine the transfection efficiency (Supplementary Figure S1). Then, KLK12 siRNA and negative control siRNA treated macrophages were infected with *M. bovis* C68004 at a multiplicity of infection (MOI) 10 without antibiotics. Then, the macrophages were incubated in 5% CO_2_ at 37 °C. After 3 h of incubation, the supernatant was removed followed by three times washing with sterile PBS to eliminate non-adherent *M. bovis*. Then, fresh RPMI 1640 (for BMDMs) and DMEM (for RAW264.7) media supplemented with FBS 10% were added and macrophages were incubated for the indicated time periods. After 48 h of transfection and then 3 h of incubation with *M. bovis*, the phagocytic ability of the BMDMs was examined (Supplementary Figure S2). For KLK12 expression experiments, BMDMs and RAW264.7 cells were treated with both strains of *M. bovis* (*M. bovis* C68004 and *M. bovis* N) with selected MOI and then incubated for indicated time points. 

### 2.7. Quantitative Real-Time PCR

Extraction of total RNA from BMDMs and RAW264.7 macrophages was performed by using the RNA extraction kit (Aidlab Biotechnology, China). The RNA integrity and concentration was measured by using NanoDrop 2000 (Thermo Scientific, Waltham, MA, USA). For cDNA synthesis, we used RevertAid First Strand cDNA synthesis kit (Thermo Scientific) by following the manufacturer’s guidelines. cDNA concentration and integrity was measured by using NanoDrop 2000 to assure the use of same amount of cDNA for each sample. For the amplification of mRNA of various genes, we used the AceQ qPCR SYBR Green Master Mix kit (Vazyme Biotech, Nanjing, China), and 700 Fast Real-Time PCR Systems (ViiA7 Real-time PCR, ABI). Normalization of expression of various genes was done by selecting β-Actin as a housekeeping gene. All the primers used in the current study are given in Table 1. The time and temperature parameters for qRT-PCR cycle were as following: 95 °C for 5 min, then 40 cycles of 95 °C for 10 s and then 60 °C for 30 s. The relative expression levels of various genes were determined as a fold change and the ΔCt values were calculated as follows: ΔCt = Ct of mRNAs − Ct of β-Actin. Then, ΔΔCt values were calculated as follows: ΔΔCt = ΔCt of treated groups − ΔCt of untreated control groups. Finally, the fold change was obtained by the 2−ΔΔCt protocol [52].

### 2.8. Detection of Apoptosis Related Proteins in Cytosolic and Mitochondrial Compartments

Purified cytosolic fraction was obtained by using the digitonin extraction method as described previously [53]. Briefly, BMDM cells were seeded into 6-well cell culture plates and transfected with KLK12 siRNA and negative control siRNA. After 48 h of transfection, the cells were infected with *M. bovis*. After 6 and 24 h of infection, cells were collected and resuspended in PBS. Cells from each well were equally divided into two aliquots; one aliquot was used for detection of total proteins. The second aliquot was resuspended in a 500 μL ice cold digitonin buffer containing 150 mM NaCl, 50 mM HEPES pH7.4 and 25 μg/mL digitonin (Sigma). The homogenates were incubated for 10 min in the ice to allow the selective membrane permeabilization. Following incubation, homogenates were centrifuged for 3 min at 980 g for three times. The pellet of the first time centrifugal was saved as the organelle fractions and the supernatants of the third time centrifugal were transferred to fresh tubes and centrifuged at 17,000 g for 10 min at 4 °C to obtain purified cytosolic fractions. Using this extraction method, no mitochondrial protein was detected in the cytosolic fraction indicating that cytosolic fraction was free of any mitochondrial contamination. Isolation of mitochondria from BMDMs was performed with the cell mitochondria isolation kit (Beyotime, Shanghai, China) according to the manufacturer’s protocol. The total and compartmental proteins were detected by the Western blot analysis by using Tubulin-α and TOM20 as loading controls for cytosolic and mitochondrial fractions, respectively. 

### 2.9. Western Blot Analysis

For WB analysis, the macrophages were washed with cold PBS twice and then homogenized by using the RIPA buffer having a mixture of phosphatase and protease inhibitors (Sigma Aldrich, St. Louis, MO, USA). The homogenized samples were placed on ice for 20 min. Later on, we sonicated the samples for 20 s and then centrifuged the samples for 20 min at 12,000× *g* at 4 °C. After centrifugation, the supernatants were taken and the SDS loading buffer (containing 250 nM Tris HCl with 6.8 pH, 0.5% bromophenol blue, 10% sodium dodecyl sulfate, 0.5 M dithiothreitol and 50% glycerol) was added. Then the samples were boiled for 10 min. Equal amounts of various proteins were separated by using 8, 10 and 12% sodium dodecyl sulfate polyacrylamide gel electrophoresis (SDS-PAGE) depending upon the size (kDa) of the proteins. The separated proteins were then transferred onto polyvinylidene difluoride (PVD) membranes (Millipore, Billerica, MA, USA). After that, membranes were blocked for one h with fresh skim milk (5%) prepared in tris buffered saline tween-20 (TBST). Then, the membranes were incubated overnight with primary antibodies at 4 °C. After this incubation, the membranes were washed for three times with TBST and then incubated with HRP-labeled secondary antibodies for one h at 37 °C. Protein bands were developed by the enhanced chemiluminescence substrate (Bio-Rad, Hercules, CA, USA) and visualized by using the BIO-RAD imaging system. The results were analyzed by using the Image J software (Version 1.50i, National Institute of Health, Bethesda, MD, USA).

### 2.10. Flow Cytometery

Apoptotic cells were assessed using an Annexin V/propidium iodide (AV/PI) staining kit according to the manufacturer’s instructions (Beyotime Biotechnology, China). Binding of Annexin V and PI was analyzed by BD FACS Calibur flow cytometer (BD Biosciences, San Jose, CA, USA). 

### 2.11. ELISA

In the cells supernatant, levels of various cytokines were measured by using ELISA kits according to the manufacturer’s instructions (Neobioscience Technology, Shenzhen, Guangdong, China). Briefly, standards and samples (100 μL each) were added into 96-well ELISA plates and incubated in a humid setting at 37 °C for 1 h and 30 min. After this, the supernatants including standards and samples were discarded and the ELISA plates were washed with a washing buffer for 4–5 times. Then, the detection antibody solution (100 μL) was added into each well and the plates were again incubated at 37 °C in a humid environment for 1 h. After that, the supernatants were discarded again and plates were washed as done previously. Then, we added HRP-conjugated antibodies (100 μL) to each well and incubated the plates at 37 °C in a humid condition for 30 min and washed the plates once again. Finally, we added the TMB substrate (100 μL) in each well and incubated the plates in the dark for 15 min at room temperature. The stop solution (100 μL) was added to each well to stop the reaction and optical density (OD) was obtained by reading the plates by the ELISA plate reader at 450 nm wavelength with a correction of 630 nm. A standard curve was obtained using two-fold dilutions of the standard for each independent experiment. Samples were added in triplicates in each independent experiment.

### 2.12. CFU Assay

To assess bacterial viability, KLK12 knockdown and negative control BMDMs and RAW264.7 macrophages were infected with *M. bovis* (MOI 10) and incubated for the indicated time periods (6 and 24 h). Thereafter, they were lysed with 0.1% Triton X 100. Appropriate dilutions were prepared for all transfected groups and plated in triplicate on Middlebrook 7H10 agar plates supplemented with mycobactin-J, sodium pyruvate and OADC. Inoculated plates were incubated at 37 °C, and colonies were counted after two weeks. 

### 2.13. Cell Viability Assay

Cell viability of the BMDMs was evaluated by the MTS tetrazolium assay. Before transfection, BMDMs were transferred into 96-well culture plates and incubated overnight. Then, the BMDMs were transfected with KLK12 siRNA and negative control siRNA as described earlier in the “Cells Transfection and Infection” section. After 48 h of transfection, the MTS reagent (20 μL) was added in each well and the BMDMs were incubated for 3 h at 37 °C in a humid environment containing 5% CO_2_. The ELISA plate reader was used to obtain the OD value at 490 nm wavelength.

### 2.14. Statistical Analysis

The data from cell experiments are representative of three independent experiments and the data are shown as mean ± SD. For comparison between the two groups, Student’s *t*-test was applied and for comparisons among more than two groups, one-way ANOVA was performed. ImageJ software (National Institute of Health, Bethesda, MD, USA) was used for the densitometric analysis of WB images while results of the flow cytometry were analyzed by the FlowJo software (Tree Star, Ashland, OR, USA). A p-value less than 0.05 reflected the findings statistically significant.

## 3. Results

### 3.1. M. bovis Infection Significantly Increases Expression of KLK12 In Vivo and In Vitro 

**Figure 1:**
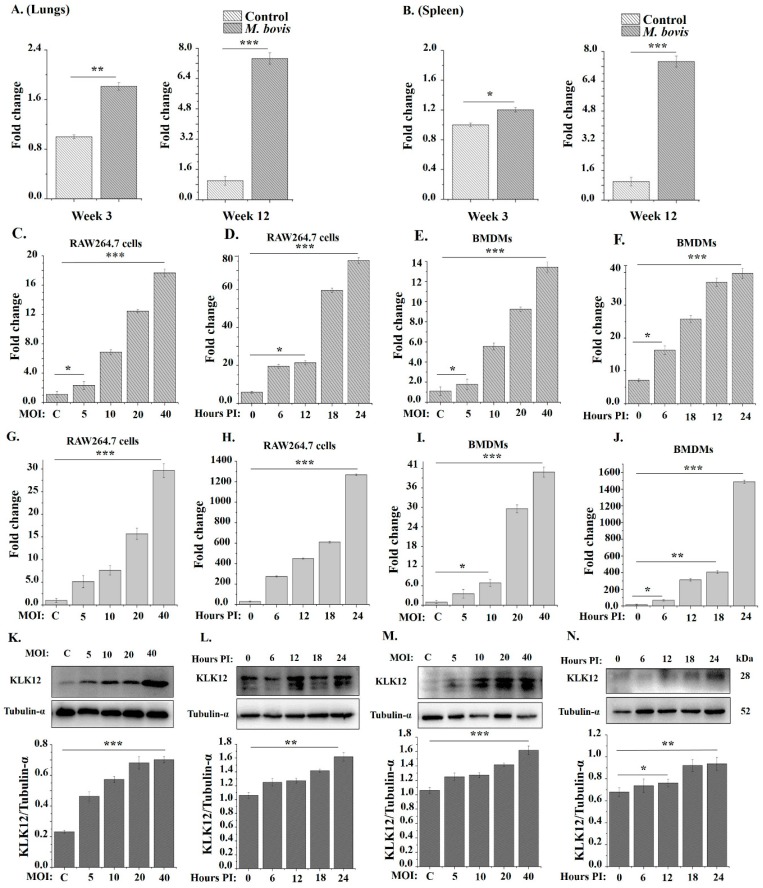
*Mycobacterium bovis* (*M. bovis*) infection upregulates KLK12 expression in mice and murine macrophages. (**A, B**) Mice (*n* = 10) were intranasally infected with *M. bovis* C68004 (at a dose of 200 CFU/mouse) or with PBS. Mice were sacrificed after 3 and 12 weeks of infection, and lungs and spleens were collected. KLK12 expression was detected by Quantitative Real-Time PCR (qRT-PCR); (**C**) RAW264.7 cells were infected with *M. bovis* C68004 at a variable multiplicity of infection (MOI) (5, 10, 20 and 40) and incubated for 24 h; (**D**) RAW264.7 cells were infected with *M. bovis* C68004 at a MOI of 10 and incubated for indicated time periods, i.e., 0, 6, 12, 18 and 24 h; (**E**) BMDMs were infected with *M. bovis* C68004 at a variable multiplicity of infection (MOI) (5, 10, 20 and 40) and incubated for 24 h; (**F**) BMDMs were infected with *M. bovis* C68004 at a MOI of 10 and incubated for indicated time periods, i.e., 0, 6, 12, 18 and 24 h; (**G**) RAW264.7 cells were infected with *M. bovis* N strain at a variable multiplicity of infection (MOI) (5, 10, 20 and 40) and incubated for 24 h; (**H**) RAW264.7 cells were infected with *M. bovis* N strain at a MOI of 10 and incubated for indicated time periods, i.e., 0, 6, 12, 18 and 24 h; (**I**) BMDMs were infected with *M. bovis* N strain at a variable multiplicity of infection (MOI) (5, 10, 20 and 40) and incubated for 24 h; (**J**) BMDMs were infected with *M. bovis* N strain at a MOI of 10 and incubated for indicated time periods, i.e., 0, 6, 12, 18 and 24 h. In all these groups, KLK12 expression was determined by qRT-PCR; (**K**) BMDMs were infected with *M. bovis* C68004 at a variable MOI (5, 10, 20 and 40) and incubated for 24 h; (**L**) BMDMs were infected with *M. bovis* C68004 at a MOI of 10 and incubated for indicated time periods (0, 6, 12, 18 and 24 h); (**M**) BMDMs were infected with the *M. bovis* N strain at a variable MOI (5, 10, 20 and 40) and incubated for 24 h; (**N**) BMDMs were infected with the *M. bovis* N strain at a MOI of 10 and incubated for indicated time periods (0, 6, 12, 18 and 24 h). KLK12 expression in these groups (K, L, M and N) was determined by the Western Blot analysis. The data showing in vitro expression of KLK12 represent the mean ± SD of three independent experiments (* *p* < 0.05; ** *p* < 0.01; *** *p* < 0.001).

To determine whether KLK12 plays a role in the immune responses against the *M. bovis* infection, we investigated the expression of KLK12 in vivo and in vitro. For in vivo determination of the KLK12 expression, we challenged C57BL/6 mice with *M. bovis* C68004. After 3 and 12 weeks of infection the mice were sacrificed and qRT-PCR results showed a significant increased expression of KLK12 in the lungs and spleen of *M. bovis* infected mice as compared to the control group (Figure 1A,B). Then, we infected murine BMDMs and RAW264.7 cells with *M. bovis* C68004 with indicated MOI. The expression of KLK12 was measured by using qRT-PCR after 24 h and it revealed an increased in the expression of KLK12 in a dose-dependent manner in both type of cells (Figure 1C,E). Further, we infected BMDMs and RAW264.7 cells with *M. bovis* C68004 at a MOI of 10 and incubated for indicated time periods, i.e., 0, 6, 12, 18 and 24 h. qRT-PCR results indicated an elevated expression of KLK12 in a time-dependent manner (Figure 1D,F). Similarly, infection of both cell types with the *M. bovis* N strain also resulted in the upregulation of KLK12 in a dose- and time-dependent manner (Figure 1G–J). Moreover, Western blot results also indicated the significant upregulation of KLK12 in BMDMs after infection with both *M. bovis* strains in a dose- (Figure 1K,M) and time-dependent manner (Figure 1L,N). These results clearly reveal that the *M. bovis* infection induces KLK12 expression in vivo and in vitro. This association of the *M. bovis* infection and KLK12 upregulation prompted us to investigate further the role of KLK12 in immune response regulation against the *M. bovis* infection. As both strains of *M. bovis* showed the same expression tendency of KLK12, we used only one strain of *M. bovis* (*M. bovis* C68004) in our further experiments. 

### 3.2. M. bovis Infection Induces Autophagy in Murine Macrophages

**Figure 2:**
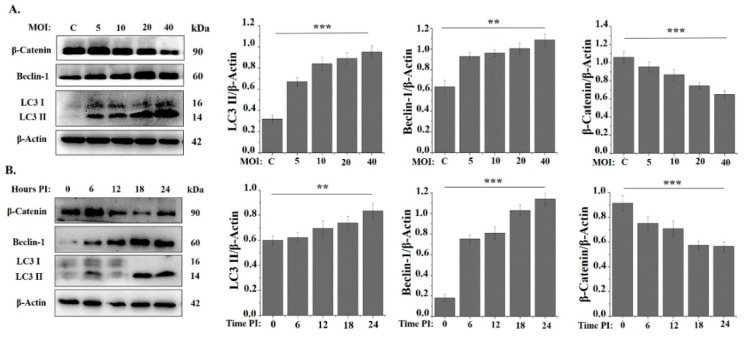
*Mycobacterium bovis* C68004 (*M. bovis*) infection induces autophagy in murine bone marrow derived macrophages (BMDMs) in a dose- (MOI) and time-dependent manner. (**A**) Murine BMDMs were infected with *M. bovis* C68004 at a variable MOI (5, 10, 20 and 40) and incubated for indicated time period. Expression of autophagic markers like LC3 II, Beclin-1 and β-Catenin was determined by the Western Blot analysis in infected and un-infected control BMDMs; (**B**) murine BMDMs were infected with *M. bovis* C68004 at a MOI of 10 and incubated for indicated time periods (0, 6, 12, 18, 24 h). Expression of autophagic markers was determined by the Western Blot analysis and expression levels of various proteins were normalized to β-Actin. Data represent the mean ± SD of three independent experiments (** *p* < 0.01; *** *p* < 0.001).

In addition to being important for cell survival and homeostasis especially during periods of limited nutrient availability, autophagy has been demonstrated to be a vital cell-autonomous defense mechanism against intracellular bacteria [54]. Therefore, we first investigated if *M. bovis* could induce autophagy in murine macrophages. Beclin-1 and LC3 play an important role in autophagy induction and are well established markers of autophagic activity [55,56]. Beclin-1 is involved in the initiation of autophagosome formation while LC3 plays a role in later phases. Under homeostatic conditions, LC3-I is localized in the cytoplasm and when autophagy is induced in various stress conditions, LC3-I is converted to LC3-II and incorporated into the autophagosome membrane. Therefore, LC3-II acts a marker of autophagy and correlates to the number of autophagosomes [57]. In the current study, we detected an increased expression of LC3-II and Beclin-1 in *M. bovis* infected macrophages. This upregulation of LC3-II and Beclin-1 was both dose- and time-dependent (Figure 2A,B). The activation of the Wnt/β-catenin signaling is also known to enhance cell survival and proliferation under various stress conditions [58]. β-catenin contains a LC3-interacting motif and thus facilitates the induction of autophagy [59]. It has been reported previously that β-catenin is degraded during the autophagic process and it may also suppress the autophagy [40,60]. Consistent with previous findings, we detected a significant decrease in the β-catenin level in a dose- and time-dependent manner in the *M. bovis* infected macrophages as compared to non-treated macrophages (Figure 2A,B). All these findings implicate that *M. bovis* induces autophagy in murine macrophages.

### 3.3. Knockdown of KLK12 Impairs Autophagy Induction in M. bovis Infected Murine Macrophages

**Figure 3:**
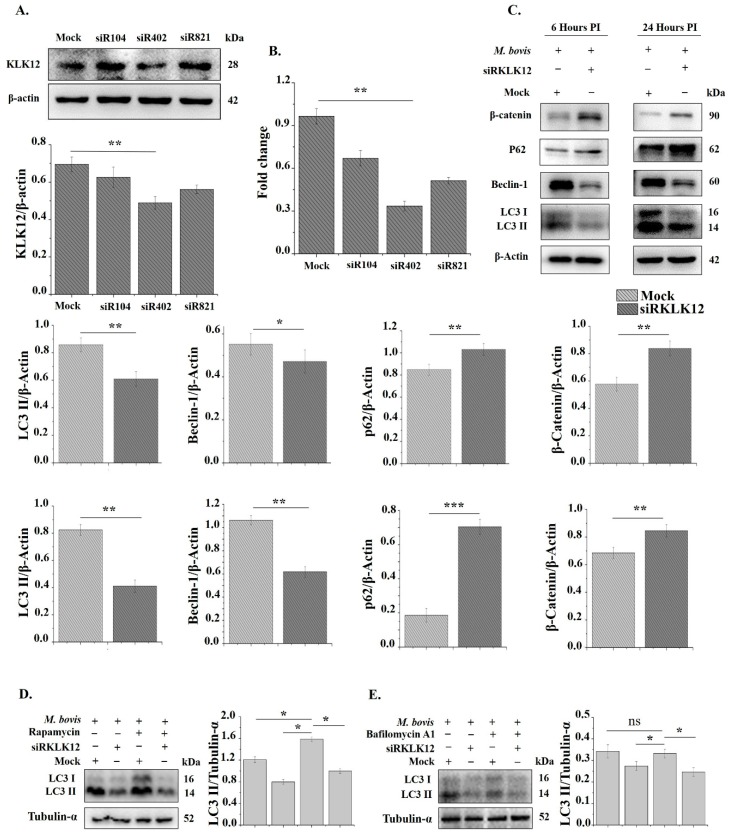
Knockdown of KLK12 impairs autophagy induction in *M. bovis* C68004 infected murine BMDMs. (**A**) Murine BMDMs were transfected with three different KLK12 siRNAs, having variable silencing targets, and a negative control siRNA (20 μM). After 48 h of transfection, KLK12 expression was determined by the Western Blot analysis. KLK12 expression was normalized to β-Actin expression; (**B**) murine BMDMs were transfected as stated above and after 48 h of transfection KLK12 expression was determined by qRT-PCR; (**C**) after 48 h of transfection, murine BMDMs were infected with *M. bovis* C68004 at a MOI of 10 and incubated for 6 or 24 h. Expression of autophagic markers LC3 II, Beclin-1, p62 and β-Catenin was determined in KLK12 siRNA and negative control siRNA transfected BMDMs by the Western Blot analysis. Expression of all the proteins was normalized to β-Actin expression; (**D**) murine macrophages were transfected as stated above and after 48 h of transfection, macrophages were treated with rapamycin (5 µm) for three h and then infected with *M. bovis* C68004 (MOI 10). After 24 h of infection, LC3 II expression was determined by WB; (**E**) murine macrophages were transfected as stated above and after 48 h, macrophages were treated with bafilomycin A1 (100 nm) for three h and then infected with *M. bovis* C68004 (MOI 10). After 24 h of infection, LC3 II expression was determined. Data represent the mean ± SD of three independent experiments (* *p* < 0.05; ** *p* < 0.01; *** *p* < 0.001).

To investigate whether KLK12 plays a role in *M. bovis* induced autophagy in murine macrophages, we produced KLK12 knockdown BMDMs by using siRNA. The knockdown efficiency of siRNAs was measured after 48 h of transfection through qRT-PCR and WB. The results showed that siRNA KLK12-mus-402 significantly downregulated the expression of KLK12 (Figure 3A,B). Therefore, we used this siRNA in our further experiments. After 48 h of transfection, BMDMs were infected with *M. bovis*. The knockdown of KLK12 resulted in the downregulation of autophagy markers LC3-II and Beclin-1 after 6 and 24 h of the *M. bovis* infection (Figure 3C). While β-catenin and p62 levels was significantly increased (Figure 3C). p62 (SQSTM1/sequestosome 1) is another autophagy marker and it also degraded during autophagy [61]. These results suggest that KLK12 plays a key role in autophagy induction. In our next experiment, we wanted to explore the signaling pathway involved in the KLK12 dependent regulation of autophagy. We further analyzed the effects of KLK12 siRNA and negative control siRNA transfection on the murine macrophage viability. BMDM and RAW264.7 cells were transfected with KLK12 siRNA and negative control siRNA and after 48 h cell viability was assessed by using the MTS assay. The siRNA transfection showed no significant difference in cell viability between the transfected groups (Supplementary Figure S3). To further validate the role of KLK12 in autophagy regulation, we detected LC3-II levels in the presence and/or absence of rapamycin (5 µm) and bafilomycin A1 (100 nm) treatments as described previously [13,62]. Rapamycin is an inhibitor of mTOR and consequently increases the autophagy [62] while bafilomycin A1 is a known inhibitor of Vacuolar H+ ATPase (V-ATPase) and it inhibits autophagic flux by preventing the acidification of endosomes and lysosomes [63,64]. We found an increased LC3-II level after the rapamycin treatment and this level was significantly reduced in siRKLK12 treated BMDMs (Figure 3D). Bafilomycin A1 affects the later stages of the autophagy and have been reported to accumulate autophagosomes/LC3 II by inhibiting the fusion between autophagosomes and lysosomes [65]. We found a non-significant effect of bafilomycin A1 on LC3-II (Figure 3E) which is also in compliance with the previous findings [13]. Taken together, these data suggest the significant role of KLK12 in *M. bovis* induced autophagy in murine macrophages.

### 3.4. KLK12 Mediated Autophagy Involves AMPK/TSC2/mTOR Signaling Pathway

**Figure 4:**
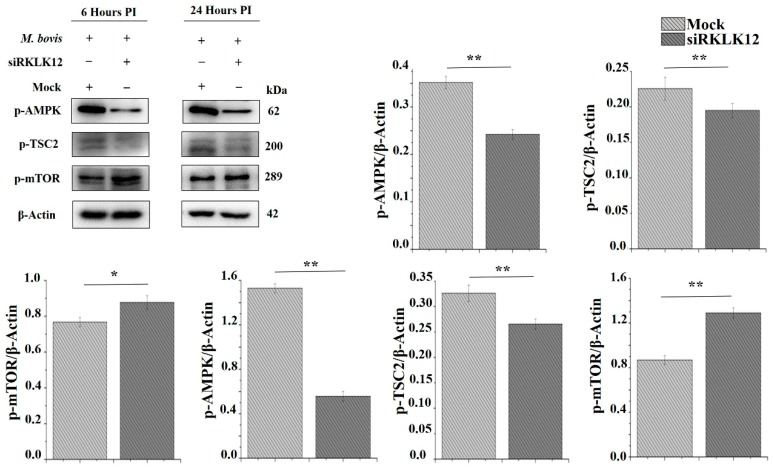
KLK12 mediated regulation of autophagy in *M. bovis* C68004 infected murine BMDMs implicates AMPK/TSC2/mTOR signaling pathway. Murine BMDMs were transfected with KLK12 siRNA and negative control siRNA (20uM). After 48 h of transfection, murine BMDMs were infected with *M. bovis* C68004 at a MOI of 10 and incubated for indicated time periods (6 and 24 h). Then, expression of phosphorylated AMPK, TSC2 and mTOR was determined in KLK12 siRNA and negative control siRNA transfected BMDMs by the Western Blot analysis. Expression of all the proteins was normalized to β-Actin expression. Data represent the mean ± SD of three independent experiments (* *p* < 0.05; ** *p* < 0.01).

The AMP-activated protein kinase (AMPK) is a multimeric serine/threonine protein kinase and the enzyme activity of AMPK is absolutely dependent on phosphorylation of the α-subunit on Thr172. It is established that AMPK has a critical role in controlling energy homeostasis in the cell [66]. It inhibits the mammalian target of rapamycin (mTOR) by phosphorylation of tuberous sclerosis complex 2 (TSC2) [67,68]. Based on these findings, we hypothesized that KLK12 mediated regulation of *M. bovis* induced autophagy in murine autophagy involves AMPK/TSC2/mTOR signaling. We infected KLK12 knockdown and negative control BMDMs with *M. bovis* for the indicated time period. The Western Blot analysis of phosphorylated AMPK, TSC2 and mTOR expression unveiled the downregulation of AMPK/TSC2/mTOR signaling in KLK12 knockdown BMDMs as compared to negative controls (Figure 4). 

### 3.5. KLK12 Regulates Apoptosis in M. bovis Infected Murine Macrophages

**Figure 5:**
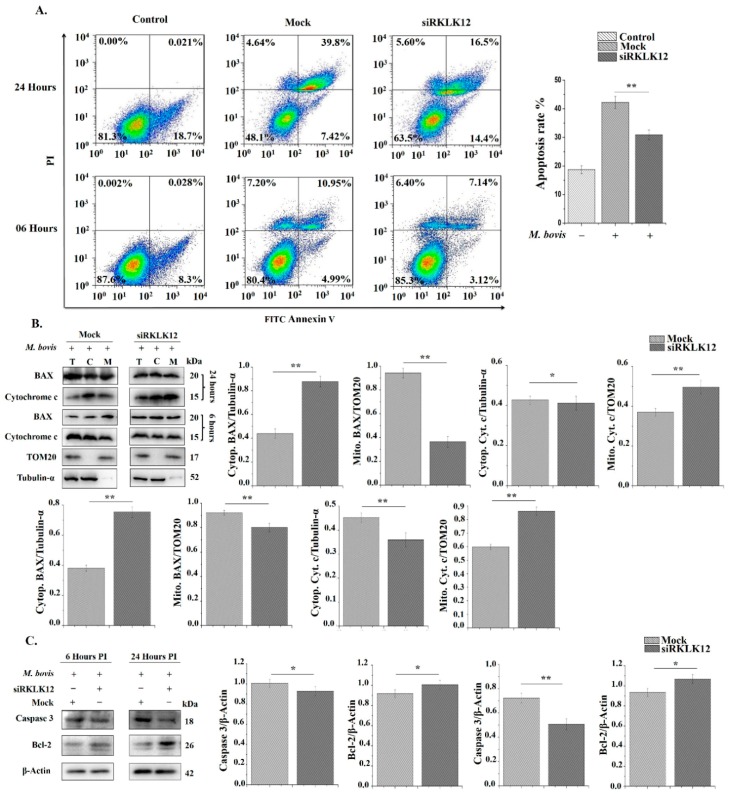
KLK12 regulates apoptosis in *M. bovis* C68004 infected murine BMDMs. (**A**) Murine BMDMs were transfected with KLK12 siRNA and negative control siRNA (20 μM). After 48 h of transfection, murine BMDMs were infected with *M. bovis* C68004 at a MOI of 10 and incubated for indicated time periods (6 and 24 h). Number of apoptotic cells was determined by Annexin V FITC and propidium iodide (AV and PI) staining according to the manufacturer’s instructions. Binding of Annexin V and PI was analyzed by the BD FACS Calibur flow cytometry (BD Biosciences, San Jose, CA, USA); (**B**) murine BMDMs were transfected with KLK12 siRNA and negative control siRNA and infected with *M. bovis* C68004 as stated above. After the indicated time periods (6 and 24 h), translocation of BAX to mitochondria and release of Cytochrome c were determined by the Western Blot analysis by using Tubulin-α and TOM20 as loading controls for cytosolic and mitochondrial compartments, respectively; (**C**) murine BMDMs were transfected with KLK12 siRNA and negative control siRNA and infected with *M. bovis* C68004. After 6 and 24 h Caspase 3 and Bcl-2 expression levels were determined by the Western Blot analysis. Expression of all the proteins was normalized to β-Actin expression. Data represent the mean ± SD of three independent experiments (* *p* < 0.05; ** *p* < 0.01). Abbreviation used: T = total; C = cytoplasmic; M = mitochondrial.

Apoptosis is an important host defense mechanism against mycobacterial infection [69,70]. Studies have reported that both autophagy and apoptosis can occur in the same cell simultaneously. Previously, we have shown that the *M. bovis* infection induces apoptosis in macrophages [71]. Therefore, we wanted to know the effect of KLK12 knockdown on apoptosis regulation in *M. bovis* infected murine macrophages. Initially, we performed flow Cytometry by using Annexin V and PI staining after 6 and 24 h of the *M. bovis* infection. The results revealed a significant decrease in the number of apoptotic cells in KLK12 knockdown macrophages (Figure 5A). B-cell lymphoma 2 (Bcl-2) family proteins are well known regulators of the apoptosis [72,73]. Some members of this family similar to Bcl-2 are anti-apoptosis whereas others such as Bax and Bak promote apoptosis [73]. In healthy cells, Bax is mainly cytosolic but has been shown to translocate to the mitochondria during apoptosis [74], whereas Bcl-2 is found at the mitochondrial membranes, endoplasmic reticulum (ER), nuclear outer membrane (NOM) and nucleus [75,76]. In apoptotic signal transduction, Bax directly targets the mitochondrial outer membrane and allows Cytochrome c to translocate into cytosol while Bcl-2 prevents Cytochrome c release [77]. After its release, Cytochrome c recruits and activates the initiator caspase-9, which subsequently activates effector caspase-3 [78,79]. Keeping in view these findings, we investigated the translocation of BAX and Cytochrome c release in the treated groups. As expected, our resulted showed a decrease in the translocation of BAX from cytosol to MOM and subsequently reduced the release of Cytochrome c from the mitochondria in KLK12 knockdown macrophages (Figure 5B). Moreover, Bcl-2 expression was upregulated in the same macrophages (Figure 5C). We further analyzed the cleaved Caspase 3 and found a decrease in its expression in KLK12 knockdown macrophages (Figure 5C). These findings implicate that KLK12 regulates the *M. bovis* induced apoptosis in murine macrophages.

### 3.6. KLK12 Regulates Cytokines Expression in M. bovis Infected Murine Macrophages

**Figure 6:**
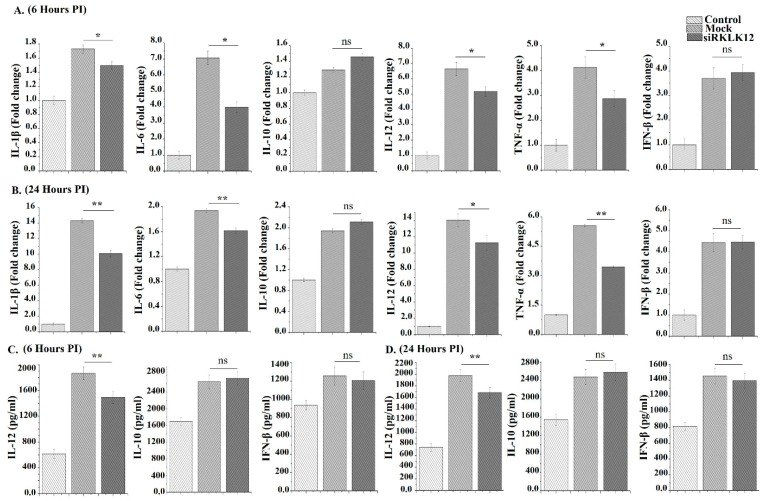
KLK12 regulates cytokines expression in the *M. bovis* C68004 infected murine BMDMs. (**A**,**B**) Murine BMDMs were transfected with KLK12 siRNA and negative control siRNA (20 uM). After 48 h of transfection, murine BMDMs were infected with *M. bovis* C68004 at a MOI of 10 and incubated for 6 (**A**) and 24 h (**B**); expression of IL-1β, IL-6, IL-12, TNF-α, IL-10 and IFN-β was determined by qRT-PCR and β-Actin was taken as internal control; (**C**,**D**) murine BMDMs were transfected with KLK12 siRNA and negative control siRNA and infected with *M. bovis* C68004 as stated above. Then, expression of IL-12, IL-10 and IFN-β were measured in cell supernatants by using ELISA kit after 6 (**C**) and 24 h (**D**) of incubation. Data represent the mean ± SD of three independent experiments (* *p* < 0.05; ** *p* < 0.01; ns, non-significant).

Cytokine expression is necessary to trigger the host cell immune response against the *M. bovis* infection and many cytokines are engaged in the regulation of autophagy [26]. So we analyzed the cytokines expression by using qRT-PCR and ELISA after 6 and 24 h of infection. The results showed a significant difference in the expression of IL-1β, IL-6, IL-12 and TNF-α between the KLK12 knockdown and negative control macrophages (Figure 6A–D). While IL-10 and IFN-β expression depicted no significant difference between the two groups (Figure 6A–D). 

### 3.7. Klk12 Promotes Antimicrobial Properties of Macrophages and Inhibits Intracellular Survival of M. bovis

**Figure 7:**
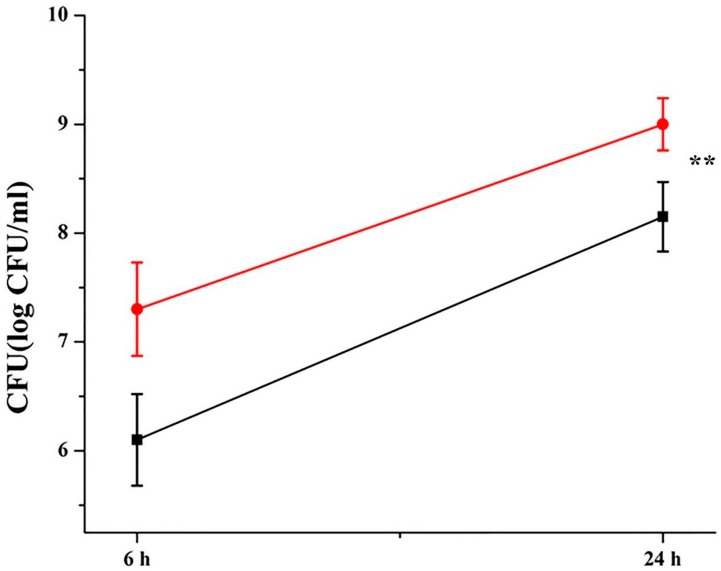
KLK12 restrains intracellular survival of *M. bovis* in murine BMDMs. Murine BMDMs were transfected with KLK12 siRNA and negative control siRNA (20 μM). After 48 h of transfection, the BMDMs were infected with *M. bovis* C68004 at a MOI of 10 and incubated for indicated times (6 and 24 h) and bacterial survival was measured by the CFU assay. Data represent the mean ± SD of three independent experiments (** *p* < 0.01).

We next investigated whether KLK12 affects the intracellular survival of *M. bovis.* To test this hypothesis, BMDMs were infected with *M. bovis* after 48 h of transfection. Knockdown of KLK12 increased the survival of *M. bovis* significantly in BMDMs after 24 h of infection while the difference in the survival rate of *M. bovis* at 6 h post infection was non-significant (Figure 7). These results indicate that KLK12 promotes the bactericidal activities of macrophages.

## 4. Discussion

Kallikreins (KLKs) are a component of the kallikrein-kinin system, which belongs to a sub-group of serine proteinases and processes low molecular weight kininogens to release kinin peptides. Many studies have reported the differential expression of various KLKs in carcinogenesis and infectious diseases [47,80]. KLK12 is one of the fifteen members of the KLKs family and a recent study illustrated the role of KLK12 in adaptability of influenza virus A to humans [81]. Moreover, the altered expression of KLK12 has been reported in many types of tumors and this expression is related with the development and metastasis of these tumors; however, the expression and role of KLK12 in the *M. bovis* infection has not been explored yet. Here, we demonstrated the increased expression of KLK12 in the *M. bovis* infection in vivo and in vitro. Previously, our lab reported the differential expression of KLK12 in the *M. bovis* infection using the microarray technique [27]. This previous study involved monocyte derived macrophages (MDMs) from *M. bovis* infected cattle. No other study has reported the KLK12 expression in the *M. bovis* infection. 

It has been reported that KLKs are involved in AMPK phosphorylation which participates in autophagy induction [82] and recently Kim et al. [45] reported that KLK6 is able to activate autophagy in various gastric cancer cell lines. Another research group investigated the involvement of KLKs in the autophagy induction under nutrient deprivation-induced stress conditions [40,41]. The study demonstrated that KLKs promote the survival of serum-starved SH-SY5Y cells via augmenting autophagy. Keeping in view these studies, we hypothesized that KLK12 may have a role in inducing autophagy in the mycobacterial infection. In the current study, the knockdown of KLK12 resulted in downregulation of autophagy in *M. bovis* infected murine macrophages. The current findings are in line with the previous related studies and it implicates that KLK12 plays an important role in the induction of immune response against the *M. bovis* infection and it affects the survival of *M. bovis* in the murine macrophages. Furthermore, the CFU analysis depicted an enhanced *M. bovis* survival in KLK12 knockdown macrophages. Previous studies also endorse that autophagy helps in restraining the intracellular growth and elimination of *M. bovis* [13,14]. AMPK is an inhibitor of mTOR and activator of autophagy. We further demonstrated that KLK12 regulation of autophagy involves the AMPK/mTOR/TSC2 pathway. Downregulation of AMPK and TSC2, and upregulation of mTOR in KLK12 knockdown macrophages suggest the involvement of this pathway in the *M. bovis* induced autophagy. The extent of autophagy induction by various mycobacterial strains differs significantly. For example, *M. smegmatis* has been reported to induce a higher autophagy response as compared to *M. bovis* bacille Calmette-Guérin (BCG) [83]. Similarly, weakly virulent and strongly virulent *M. tuberculosis* strains exhibit a variable induction of autophagy and apoptosis as reviewed recently [84]. Virulent strain, *M. tuberculosis* H37Rv, is able to limit the autophagy by upregulating the host anti-autophagic factor Bfl-1/A1 while *M. tuberculosis* H37Ra, avirulent strain, enhances the autophagic response [85]. *M. indicus pranii* (MIP) is a non-pathogenic mycobacterium which induces immune response and upregulates autophagy reviewed in [86]. MIP has been used as a promising immuno-modulator against *M. tuberculosis* in the guinea pig model of TB [87]. Keeping in view the importance of autophagic response, several innovative TB therapies based on autophagy manipulation have been proposed and some of them have a high potential for future clinical trials and implementation in healthcare systems.

Several reports have shown that autophagy and apoptosis can occur in the same cell and apoptosis regulators can control autophagy and autophagy can also contribute to apoptosis in tissue homeostasis, development and disease [88]. In the current study, the flow cytometry and WB analysis revealed the downregulation of apoptosis in the KLK12 knockdown *M. bovis* infected macrophages as compared to controls indicating a positive role of KLK12 in regulation of apoptosis. Earlier studies have shown that the *M. bovis* infection induces apoptosis in macrophages [71] but there is no report on the correlation of KLK12 expression and apoptosis in the *M. bovis* infection. However, a few previous studies unveiled that serine proteases are able to mediate apoptosis-like cell death and phagocytosis [89,90]. In a recent study on cancerous cell lines, Li and He [91] found a reduced cell proliferation in the KLK12 knockdown AGS cells. Similarly, the knockdown of KLK11 has been reported to enhance the apoptosis of colorectal cancer cells [92]. The anti-apoptotic function of KLK1 in renal candidiasis has been described. It was noticed that the KLK1 expression is IL-17-dependant and it enhances the cell survival. Overexpression of KLK1 or treatment with bradykinin salvaged *IL-17RA^-^/^-^* mice from renal candidiasis [93]. The protective role of KLK1 in renal injury has been reported by other research groups [94]. In a previous study, researchers using murine splenocytes and the human Jurkat T cell line demonstrated that KLK6 strongly supports cell survival. KLK6 over expression and recombinant KLK6 was shown to significantly reduce cell death under resting conditions and in response to camptothecin, dexamethasone and staurosporine [95]. The findings of these studies are not in compliance with the current study due to variation in the cell type used, and difference in the disease and type of KLK under investigation. To further examine the mechanism underlying the downregulation of apoptosis, we investigated the translocation of cytosolic BAX to mitochondria and the release of Cytochrome c from mitochondria. We also evaluated the expression of Bcl-2 as it is also a key protein of the apoptosis signaling pathway [96]. The Western blot analysis depicted a significant reduction in the translocation of cytosolic BAX and release of Cytochrome c in the KLK12 knockdown murine macrophages while an increase in Bcl-2 expression was noticed in the same macrophages. Caspase-3, which is an essential mediator of apoptosis and frequently activates death proteases [97] was also downregulated in the current study. Collectively, these findings indicate that KLK12 serves a crucial function in the cell proliferation and apoptosis. 

Innate immunity relies essentially on the behavior of inflammatory mediators. Pro-inflammatory cytokines similar to TNF-α and IL1 play an important role in the innate immune response against invading *M. bovis* [98]. It is established that the *M. bovis* infection of macrophages leads to altered expression of cytokines [99,100]. In the current study, we wanted to know if the KLK12 knockdown has any effect on cytokine expression in *M. bovis* infected macrophages. We used qRT-PCR and ELISA to determine this expression. The results showed a significant downregulation of IL-1β, IL-6, IL-12 and TNF-α in KLK12 knockdown macrophages while IL-10 and IFN-β had no significant difference of expression between the two groups. Many other studies have investigated the expression and role of cytokines in *M. bovis* and other mycobacterial species infected macrophages [101,102]. But there is no study elaborating the role of KLK12 in the cytokine regulation. In a related study, researchers reported that KLK1 activates pro-inflammatory pathways in proximal tubular epithelial cells of kidney under the diabetic milieu [103]. Recombinant KLK1 increased the production of inflammatory cytokines through the activation of p42/44 and p38 MAPK signaling pathways while the knockdown of endogenous KLK1 expression mitigated IL-8 and ICAM-1 productions in vitro. This study also suggested the KLKs regulation of cytokine production in stress conditions. 

In summary, the current study for the first time demonstrated the augmented expression of KLK12 and validated its protective role in immune response modulation against *M. bovis* infection. Interestingly, most of the previous studies remained focused on the expression and role of KLKs under benign and malignant conditions with a negligible number of studies elaborating the expression and role of KLKs in degenerative and infectious diseases. Our findings supported our hypothesis about the expression and role of KLK12 in immune response modulation in the *M. bovis* infection. KLK12 expression was upregulated in vivo and in vitro after the *M. bovis* infection. The knockdown of KLK12 significantly impaired the *M. bovis* induced autophagy in murine macrophages. Our findings further suggest that this KLK12 regulation of autophagy involves the AMPK/mTOR/TSC2 pathway. In a previous study, KLKs mediated autophagy induction has been attributed to activation of p38 mitogen-activated protein kinases (p38 MAPK) similar to MEK1/2/ERK1/2 [41]. Similarly, KLK6 has been shown as a autophagy-related gene and it depends on p53 gene activation to induce autophagy in gastric cancer cell lines [45]. Further studies may unveil the role of these signaling pathways in the KLK12 regulation of autophagy. Apoptosis is also a part of host innate defense mechanisms. The results of the current study implicate that KLK12 also have a role in apoptosis regulation. Moreover, the knockdown of KLK12 by siRNA altered the pro-inflammatory cytokines including IL-1β, IL-6, IL-12 and TNF-α. All these cytokines are actively involved in modulating innate immune response. Previous studies have reported that KLKs mediated the regulation of cytokine and apoptosis involves activation of bradykinin receptor 1 and 2 (BIR/B2R) and also the upregulation of ERK1/2 [104]. Therefore, it is likely that these pathways are also involved in the KLK12 regulation of apoptosis and cytokine expression in the *M. bovis* infection. In the end, we report that by the modulation innate immune response, KLK12 also contributes to restrain the intracellular growth of *M. bovis*. This study highlights a different and new aspect of host and pathogen interaction in the mycobacterial infection. Further investigations may open a new avenue for diagnosis and host-directed therapy of human and bovine TB.

## Figures and Tables

**Table 1:** Primers used in the present study.

Gene Name	Forward Primer (5′-3′)	Reverse Primer (5′-3′)
*B-Actin*	5-TGTTACCAACTGGGACGACA-3	5-ACCTGGGTCATCTTTTCACG-3
*KLK12*	5-CAGCCAGACTCTCTGGTTCC-3	5-TCCAGCCCCTAGCTAACAGA-3
*IL-1β*	5-AAGGAGAACCAAGCAACGACAAAATA-3	5-TTTCCATCTTCTTCTTTGGGTATTGC-3
*IL-6*	5-CCCAATTTCCAATGCTCTCCTA-3	5-AGGAATGTCCACAAACTGATATGCT-3
*IL-10*	5-AGCATTTGAATTCCCTGGGTGA-3	5-CCTGCTCCACTGCCTTGCTCTT-3
*IL-12*	5-CCAAATTACTCCGGACGGTTCAC-3	5-CAGACAGAGACGCCATTCCACAT-3
*TNF-α*	5-AGAGCTACAAGAGGATCACCAGCAG-3	5-TCAGATTTACGGGTCAACTTCACAT-3
*IFN-β*	5-AAGAGTTACACTGCCTTTGCCATC-3	5-CACTGTCTGCTGGTGGAGTTCATC-3

## Supplementary Materials

The following are available online at https://www.mdpi.com/2073-4409/8/5/415/s1, Figure S1: Examination of transfection efficacy, Figure S2: Evaluation of phagocytic ability of BMDMs after transfection Figure S3: Assessment of cell viability, Table S1: Sense- and antisense sequences of KLK12 siRNA, negative control siRNA and FAM negative control siRNA.
