# Supplementary material for: Kallikrein 12 Regulates Innate Resistance of Murine Macrophages against Mycobacterium bovis Infection by Modulating Autophagy and Apoptosis
Source: Cells. 2019 May 5;8(5):415. doi: 10.3390/cells8050415 (PMC6562459; doi:10.3390/cells8050415)
Supplement: Supplementary file 1 [file cells-08-00415-s001.pdf]

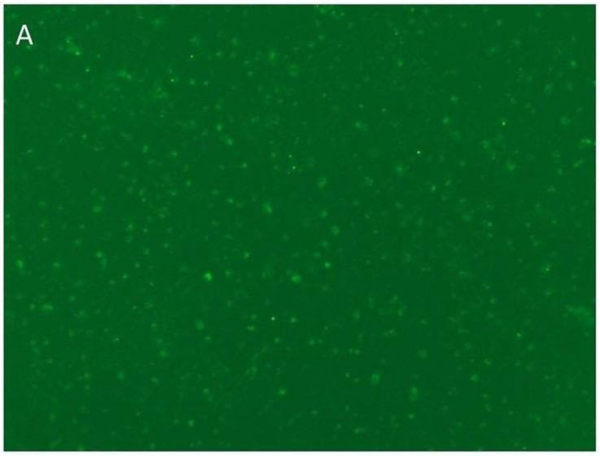

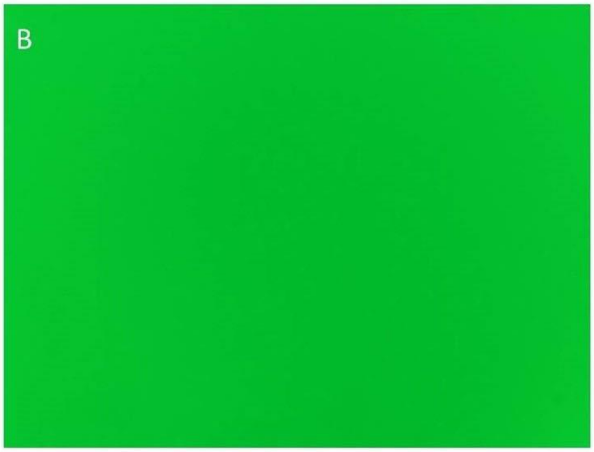

Supplementary Figure 1. Murine BMDMs were transfected with FAM negative control siRNA (20uM). After 48 hours of transfection, macrophages were observed under fluorescent microscope to examine the transfection efficiency and were compared with non-FAM treated negative controls**.**

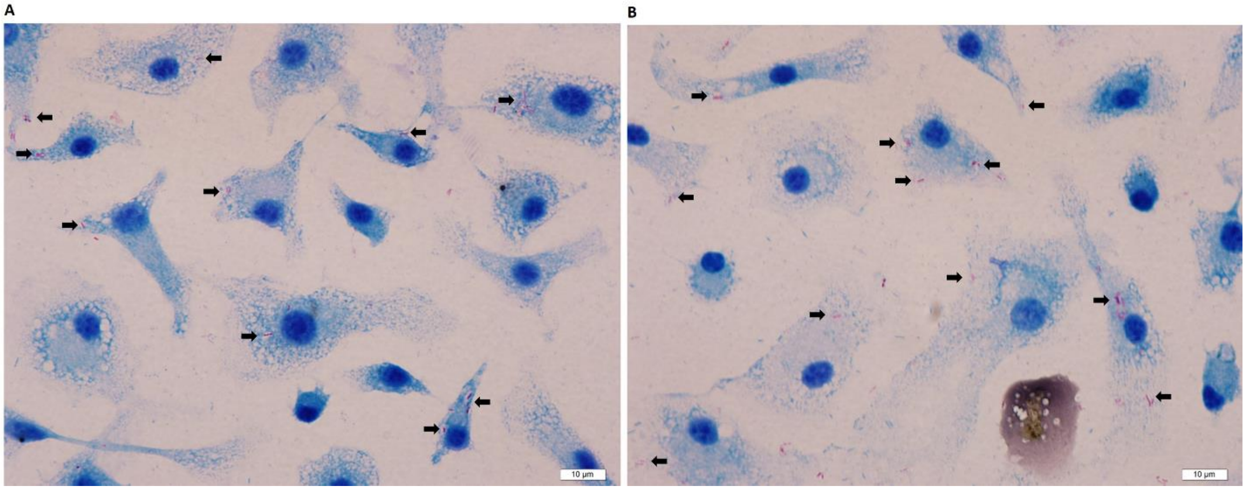

Supplementary Figure 2. Murine BMDMs were transfected with KLK12 siRNA (A) and negative control siRNA (B). After 48 hours of transfection, the macrophages were infected with *M. bovis* C68004 at a multiplicity of infection (MOI) 10. Following incubation for 3 h at 37°C in 5% CO2, the supernatant was discarded and each well was washed three times with sterile PBS to remove non-adherent *M. bovis*. After washing and fixation, the macrophages were stained with Acid Fast staining according to the manufacturer’s instructions (Solarbio Science and Technology, Beijing). Phagocytized bacilli were observed under 100X.

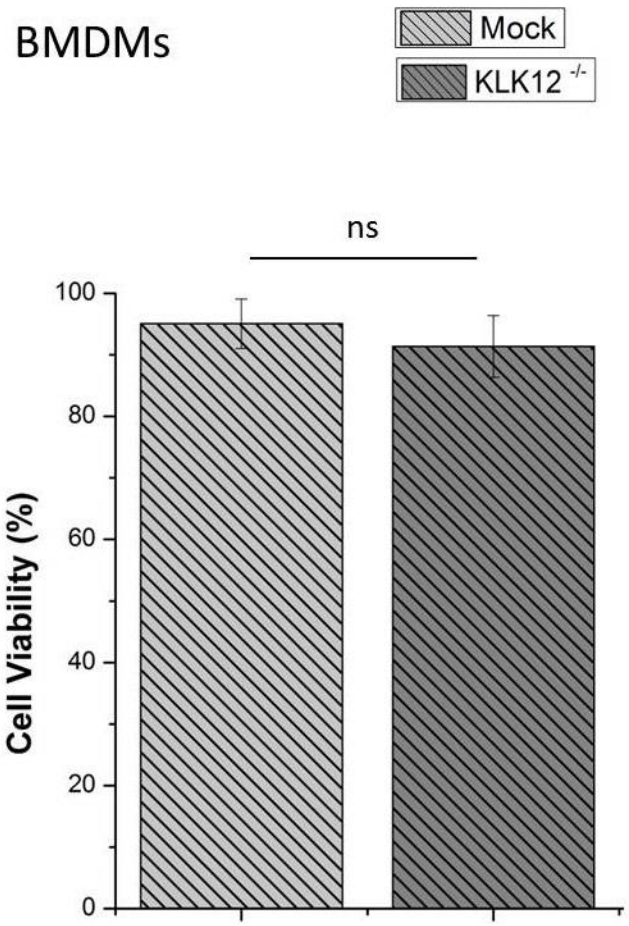

Supplementary Figure 3. BMDMs were cultured in 96-well plates overnight before transfection with KLK12 siRNA and negative control siRNA. After 48 hours of transfection, 20 µl MTS reagent was added in each well and macrophages were incubated at 37°C for 3 h in a humidified, 5% CO2 atmosphere. OD value was determined by measuring the absorbance at 490 nm wavelength with an ELISA plate reader.

Supplementary Table 1 Sense- and antisense sequences of KLK12 siRNA, negative control siRNA and FAM negative control siRNA

S. No.	siRNA	Sense (5´-3´)	Antisense (5 ´-3´)
1.	KLK12-mus-104	GGUUCAGCAUUCUCUUGCUTT	AGCAAGAGAAUGCUGAACCTT
2.	KLK12-mus-402	UCAGAACCAUGAGCAUGAUTT	AUCAUGCUCAUGGUUCUGATT
3.	KLK12-mus-821	GAAUAGUCAUCAGGAAUAATT	UUAAUUCCUGAUGACUAUUCTT
4.	Negative Control	UUCUCCGAACGUGUCACGUTT	ACGUGACACGUUCGGAGAATT
5.	Negative Control FAM	UUCUCCGAACGUGUCACGUTT	ACGUGACACGUUCGGAGAATT
